# Supplementary material for: Complex‐centric proteome profiling by SEC‐SWATH‐MS
Source: Mol Syst Biol. 2019 Jan 14;15(1):e8438. doi: 10.15252/msb.20188438 (PMC6346213; doi:10.15252/msb.20188438)
Supplement: Supplementary file 8 — Dataset EV7 [file MSB-15-e8438-s008.zip › feature_plots_string/Q96ES7.pdf]

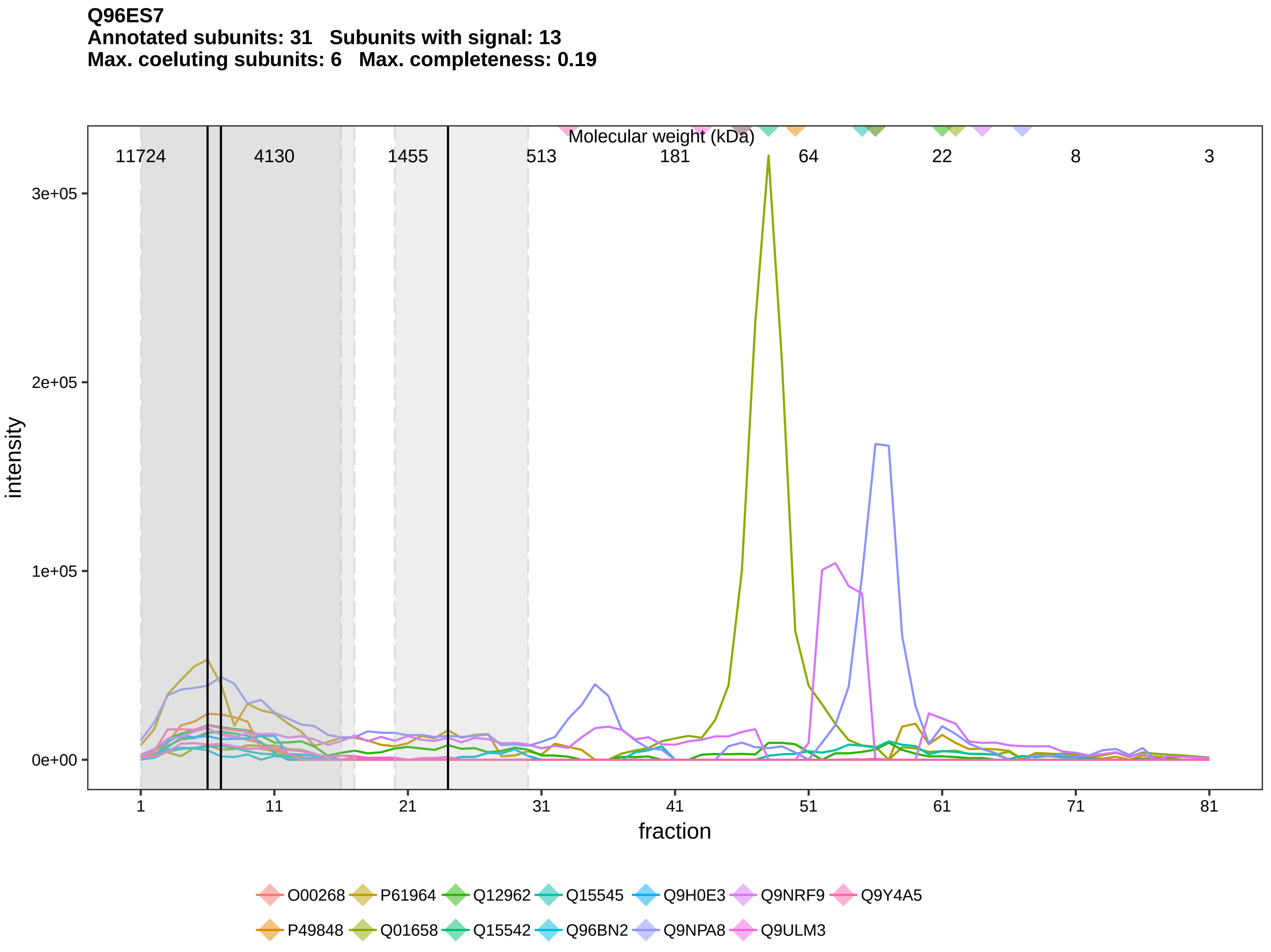
Q96ES7
Annotated subunits: 31 Subunits with signal: 13
Max. coeluting subunits: 6 Max. completeness: 0.19
Molecular weight (kDa)
11724
4130
1455
513
181
64
22
8
3
3e+05
2e+05
1e+05
0e+00
intensity
1
11
21
31
41
51
61
71
81
fraction
O00268
P61964
Q12962
Q15545
Q9H0E3
Q9NRF9
Q9Y4A5
P49848
Q01658
Q15542
Q96BN2
Q9NPA8
Q9ULM3
